# Controversies Surrounding Vitamin D: Focus on Supplementation and Cancer

**DOI:** 10.3390/ijerph16020189

**Published:** 2019-01-11

**Authors:** Salvatore Minisola, Federica Ferrone, Vittoria Danese, Veronica Cecchetti, Jessica Pepe, Cristiana Cipriani, Luciano Colangelo

**Affiliations:** Department of Internal Medicine and Medical Disciplines, “Sapienza” Rome University, 00161 Rome, Italy; federica.ferrone@uniroma1.it (F.F.); vitto_danese@yahoo.it (V.D.); veronica.cecchetti@uniroma1.it (V.C.); jessica.pepe76@gmail.com (J.P.); cristiana.cipriani@gmail.com (C.C.); luciano.colangelo@hotmail.it (L.C.)

**Keywords:** vitamin D, supplementation, cancer incidence, cancer mortality

## Abstract

There has recently been a huge number of publications concerning various aspects of vitamin D, from the physiological to therapeutic fields. However, as a consequence of this very fast-growing scientific area, some issues still remain surrounded by uncertainties, without a final agreement having been reached. Examples include the definitions of vitamin D sufficiency and insufficiency, (i.e., 20 vs. 30 ng/mL), the relationship between 25-hydroxyvitamin D (25(OH)D) and parathyroid hormone, (i.e., linear vs. no linear), the referent to consider, (i.e., total vs. free determination), the utility of screening versus universal supplementation, and so on. In this review, the issues related to vitamin D supplementation in subjects with documented hypovitaminosis, and the role of vitamin D in cancer will be concisely considered. Daily, weekly, or monthly administration of cholecalciferol generally leads to essentially similar results in terms of an increase in 25(OH)D serum levels. However, we should also consider possible differences related to a number of variables, (i.e., efficiency of intestinal absorption, binding to vitamin D binding protein, and so on). Thus, adherence to therapy may be more important than the dose regimen chosen in order to allow long-term compliance in a sometimes very old population already swamped by many drugs. It is difficult to draw firm conclusions at present regarding the relationship between cancer and vitamin D. In vitro and preclinical studies seem to have been more convincing than clinical investigations. Positive results in human studies have been mainly derived from post-hoc analyses, secondary end-points or meta-analyses, with the last showing not a decrease in cancer incidence but rather in mortality. We must therefore proceed with a word of caution. Until it has been clearly demonstrated that there is a causal relationship, these positive “non-primary, end-point results” should be considered as a background for generating new hypotheses for future investigations.

## 1. Introduction

During the past decades, there has been an impressive number of publications concerning various aspects of vitamin D. These mainly include (but are not only restricted to) both physiological and therapeutic aspects. However, despite this florid scientific literature, little certainty, and therefore, consensus exist on many issues. A short list of the most important controversies surrounding the vitamin D field is reported in Table 1.

It is clear that addressing all aspects of vitamin D is impossible because each item has been extensively debated with arguments in favor or against. Therefore, I will concisely focus on two aspects mainly because of personal experience (i.e., pharmacological supplementation), and possible interest for future applications (i.e., the role of vitamin in cancer prevention). For the remaining issues, the reader can refer to some relevant reviews recently published that deeply cover these arguments [1,2].

## 2. Supplementation in Normal Subjects

One of the most significant achievements in the history of medicine was the discovery in 1919 that sunlight was able to cure rickets; later, vitamin D was identified. Solar ultraviolet B radiation (UVB) is now considered the main source of vitamin D, representing an easy way to reach adequate levels of 25-hydroxyvitamin D (25(OH)D) values in serum at no cost. However, government bodies, especially in the United States, are concerned about the possibility that this exposure, if uncontrolled, may promote skin cancer. Accordingly, the United States Preventive Services Task Force (USPSTF) issued recommendations on behavioral counseling for skin cancer prevention. USPSTF concluded that “behavioral interventions can increase sun protection behavior, but there is no consistent evidence that interventions are associated with a reduction in the frequency of sunburn in children or adults and minimal evidence on skin cancer outcomes”. However, the task force concluded that clinicians should counsel children and young adults with fair skin types to minimize exposure to UV radiation (grade B recommendation, i.e., there is high certainty that the net benefit is moderate, or there is moderate certainty that the net benefit is moderate to substantial), and that there may be a small benefit of counseling adults at high risk for skin cancer about minimizing UV exposure (grade C recommendation, i.e., there is at least moderate certainty that the net benefit is small)” [3]. From a practical point of view, people with type II skins (fair white) living, for example, at 40° latitude may get their needs of vitamin D by spending about 15 min in the sun exposing face, arms, and legs 2–3 times weekly, between 11.00 a.m. and 3.00 p.m., during the period of the year going from May through to October [4]. People with darker skin are required to spend more time in the sun. People with type I skin (very fair white) require less time spent in the sun; they also have a higher risk of non-melanoma skin cancer. In any case, people should avoid sunburn at any age, because the association with the increased risk of melanoma and non-melanoma skin cancer is fairly well-established. The possibility of producing vitamin D decreases proportionally as latitude increases. The contrary is observed with a decreasing degree of latitude, so that at the equator you have the possibility of producing vitamin D throughout the year [5].

Having said that, according to some authorities in the field, sufficient non-burning sun exposure can be allowed to reach the desired threshold of 25(OH)D [6]. However, the large majority of people and doctors are scared of the connection between UVB and skin cancer. At the same time, the prevalence of hypovitaminosis D is of pandemic proportion [7], so that other possible forms of vitamin replenishment should be encouraged.

Following the observation that sunlight cures rickets, another important step forward in the history of vitamin D was made in 1924, when it was found that an inactive lipid in the diet and skin could be converted by UV light into an anti-rachitic substance [8]. It was therefore logical to believe that a sufficient vitamin D status could be reached by eating food containing adequate amounts of vitamin D. Unfortunately, foods containing this steroid are not frequently consumed in the world, apart from some specific areas of the continent. Indeed, vitamin D_2_ can be found mainly in wild mushrooms. Beef and dairy products contain vitamin D_2_ and 25(OH)D_2_. Vitamin D_3_ and 25(OH)D_3_ are found in fish, eggs, meat, and dairy products. If we consider the amount of vitamin D per 100 g of weight, the best sources of vitamin D are represented by cod liver oil (90–250 mcg), fatty fish like salmon (6–10 mcg), and wild mackerel (5–8 mcg) [9].

There are many studies that have investigated the prevalence of low 25(OH)D concentrations in various parts of the world. However, data reporting dietary vitamin D intake are scanty. As far as this last point is concerned, a study reported a median vitamin D intake below 5 mcg (200 I.U.) per day in the majority of the countries in the world [10]. Evaluating both D_2_ and D_3_, and their respective 25-hydroxy metabolites in foods is not an easy task, since, among other things, food composition varies frequently. An updated list of selected food sources of vitamin D can be found at https://ods.od.nih.gov/factsheets/VitaminD-HealthProfessional/.

In this context, another possibility for tackling the problem of pandemic hypovitaminosis D is represented by food fortification or, alternatively, adopting a vitamin D biofortification strategy. A Vitamin D biofortification strategy is defined as a modality of increasing the total vitamin D content of foods of animal origin, by increasing the dietary vitamin D or 25(OH)D amount in animal feed [11]. Such new strategies could be advantageous to increasing the vitamin D levels in the world. We are at the very early stages of these new modalities of overcoming the problem, and, at least in Europe, different recommendations have been adopted within countries concerning these types of interventions. The most important example of food fortification comes from Finland, where systematic voluntary vitamin D food fortification was introduced in 2003 by adding a dose of 10 mcg/100 g to all fat spread, and a dose of 0.5 mcg/100 g to all fluid milk products; the amounts were doubled in 2011. As a result of this supplementation, mean serum 25(OH)D values increased from about 19 ng/mL in 2003 to about 26 ng/mL in 2011 [12]. In addition to other western countries (United States and Canada), vitamin D fortification has been also implemented in other nations such as India and Jordan. Details of biofortification, including trials and implementations in the world, is extensively covered in [13].

The third option to increase serum 25(OH)D levels is represented by pharmacological supplementation. The theory behind this approach can be reviewed in [14]. Regarding pharmacological supplementation, there has been a long debate concerning optimal vitamin D supplementation strategies [15,16,17,18]. There is, for the time being, a consensus that the so-called “stoss therapy” (that is, large single doses of oral vitamin D) should be avoided as it increases the risk of falls, and therefore the incidence of fractures. However, the pathophysiological mechanism by which huge doses of cholecalciferol increase falls is only hypothesized, but has never been clearly demonstrated [17,18]. In this context, it should be noted that in humans, bolus doses greater than 150,000 I.U. are rarely needed [14], unless subjects are severely deficient. Along these lines, 100,000–125,000 I.U. of vitamin D have been administered at the beginning of important trials evaluating the effect of antiresorptive drugs on fracture [19] to the end of rapidly obtaining optimal vitamin D status.

Table 2 reports some of the most important recent studies addressing the problem of reaching vitamin D sufficiency in normal subjects, with documented hypovitaminosis D. When analyzing these investigations, the conclusion seems that daily, weekly, or monthly administration of vitamin D (cholecalciferol) generally leads to essentially similar results, even though the monthly dosing had the lowest average 25(OH)D increase of about 121% compared to basal values. However, we should also consider possible differences related to a number of variables, (i.e., efficiency of intestinal absorption, binding to vitamin D binding protein, and so on). With this background, we believe that the adherence to therapy may be more important than the dose regimen chosen, in order to allow long-term compliance in sometimes very old populations already swamped by many drugs.

## 3. Vitamin D and Cancer

An increasing number of papers have been published concerning possible links between vitamin D and cancer. This has been mainly driven by the notion that CYP27B1, the enzyme that converts 25(OH)D to its final metabolite, as well as VDR (vitamin D receptor), are present not only in the kidneys but also in extra-renal sites [20,21,22,23]. These may include tumor cells, and cells of the tumor microenvironment. Furthermore, the relatively low cost of vitamin D and its derivative in respect to classical and new (i.e., biological) treatments, renders this form of therapy appealing. This section briefly reports the most recent in vitro, preclinical, and clinical studies detailing possible links between native vitamin D and cancer.

### 3.1. Preclinical Studies

Recently, more sophisticated in vitro and preclinical studies seemed to strongly suggest a role for vitamin D in modulating or even halting tumor growth. For example, Liu and coworkers demonstrated that the delivery of vitamin D-based drug payloads via tumor-targeted epidermal growth factor receptor (EGFR) liposomal nanoparticles has promise as a new therapy of EGFR tyrosine kinase inhibitors for resisting lung cancer [24]. Feldman and coworkers demonstrated [25] that vitamin D treatment is able to: (1) decrease insulin resistance, (2) reduce leptin, (3) increase adiponectin signaling and (4) regulate the serine–threonine liver kinase–B1–AMP activated protein kinase (LKB–AMPK) pathway. All these actions contribute to an overall decrease in local estrogen synthesis in obese mice, thus mitigating obesity-enhanced breast cancer cell growth in the postmenopausal period. The same group also demonstrated that in breast cancer cells VDR regulation of the inhibitor of differentiation 1 (ID1) expression is mechanistically conserved in tumors. They also found a negative association between circulating 25(OH)D levels and the expression of ID1 in primary tumors from breast cancer cells. These data point to the possible beneficial effect of correcting hypovitaminosis D on breast cancer progression [26].

### 3.2. Clinical Studies

Do the preclinical data find confirmation in human studies? The strongest evidence to date has come from observational studies on serum 25(OH)D concentrations. Indeed, so-called “geographic ecological” studies have found inverse correlations between indices of solar ultraviolet B radiation and the incidence and/or mortality rates of about 20 cancers [27].

Compared with previous investigations, three recent clinical studies seem to better characterize the role of cholecalciferol, at least in breast and colon cancer. In the first study, Lappe and coworkers examined the effect of dietary supplementation with calcium and vitamin D_3_ on the risk of developing cancer [28]. Healthy postmenopausal older women with mean serum 25(OH)D levels of 32.8 ng/mL were enrolled. Compared with placebo, this supplementation did not result in a significantly lower risk of all type cancer at 4 years. However, in a post hoc analysis, the achieved serum 25(OH)D level was significantly inversely associated with cancer incidence (*p* = 0.03). Compared with 25(OH)D level of 30 ng/mL as baseline, the estimated HR for cancer incidence for 25(OH)D levels between 30 ng/mL and 55 ng/mL was 0.65 (95% CI, 0.44–0.97). Other factors might have obscured the negative effect in the population as a whole, including the fact that sample size calculations were based on a study cohort with lower baseline 25(OH)D levels, thus limiting the power to find an effect of vitamin D_3_ supplementation. Thus, and most importantly, the null finding of this study might reflect that most participants were already replete in vitamin D at study entry.

The same group later carried out a study to investigate the possible relationship between 25(OH)D concentration and breast cancer risk across a broad range of 25(OH)D concentrations among women aged 55 years or older [29]. They used data from two randomized clinical trials [28,30] and one prospective cohort [29], reaching a total number of 5038 postmenopausal women. Using three different statistical methods, they found that higher 25(OH)D concentrations were associated with a dose–response decrease in breast cancer risk, with concentrations ≥60 ng/mL being most protective.

Finally, McCullough and coworkers [31] pooled participant-level data from 17 cohorts, comprising 5706 colorectal cancer case participants and 7107 control participants, over a wide range of circulating 25(OH)D levels to identify optimal concentrations for colorectal cancer risk reduction. Colorectal cancer risk decreased steadily and in a statistically significant manner with increasing pre-diagnostic circulation 25(OH)D up to 40 ng/mL (in the population as a whole). When the data were analyzed by sex, they found that high concentrations of 25(OH)D levels were related to a statistically significant lower colorectal cancer risk only in women. They conclude that the optimal 25(OH)D levels for this type of cancer reductions are in the range of 30–40 ng/mL.

The results of these studies therefore suggest that at least for some cancer types, the ideal values are not those recommended by the Institute of Medicine, now the National Academy of Medicine, which is 20 ng/mL. They are also in accordance with the hypothesis that thresholds for vitamin D can differ according to the parameter or condition chosen. We are aware of studies reporting negative results. The most relevant among these investigations was by the Women’s Health Initiative (WHI) [32]. This trial did not find an association between the assigned vitamin D treatment group and breast cancer risk. However, it should be noted that the amount of vitamin D administered was only 400 I.U./day, and the estimated compliance was very poor (about 50%). A subsequent re-analysis of the WHI data showed a significant reduction in breast cancer risk among women not taking vitamin D or calcium supplement before enrollment, thus emphasizing the protective effects [33]. The same considerations can be made concerning colorectal cancer risk in the frame of WHI. In addition to the issues of dose and compliance, the absence of pre- and post-intervention 25(OH)D measurements preclude an appropriate interpretation of the data. Also in this case, a reanalysis of the WHI data indicated that supplemental vitamin D and calcium were associated with lower colorectal cancer risk only among women not randomly assigned to receive exogenous estrogen [34], possibly due to the effect of estrogen on vitamin D activity.

Following the initial submission of this manuscript, two papers were published online addressing the issue of the possible prevention of cancer by vitamin D. In the first, published in the New England Journal of Medicine [35], 25,871 subjects were administered cholecalciferol at a dose of 2000 I.U. per day (and marine n-3 fatty acids at a dose of 1.0 g. per day, for possible cardiovascular disease prevention), and were followed for a median of 5.3 years. Supplements with vitamin D did not cause a lower incidence of invasive cancer (or cardiovascular disease) in comparison with a placebo. However, also for this trial, a post hoc analysis of the rate of death from cancer suggested a possible benefit with respect to the rate of total cancer after the exclusion of early follow-up data. This secondary analysis was theoretically sound, at least from a clinical point of view, owing to the expected latency of the effect of vitamin D in preventing cancer. The second paper [36] was a post hoc analysis of data from a vitamin D assessment (VIDA) study that included 5108 participants from family practitioners and community groups in New Zealand. Subjects were treated with cholecalciferol (initial bolus of 200,000 I.U., followed by monthly doses of 100,000 I.U. or placebo), and were followed for a median period of 3.3 years. The post hoc primary outcome—the number of all primary invasive and in situ malignant cancers diagnosed from randomization until the study medication was discontinued—was not satisfactory. It should be noted that, as the authors admit, the study had 85% power to detect a risk ratio of 0.70, as reported in Lappe’s study [28]; however, the power was much lower for detecting an association between cancer and vitamin D.

It is difficult to draw firm conclusions at present regarding the relationship between cancer and vitamin D. In vitro and preclinical studies seem to have been more convincing than clinical investigations. For example, it has been shown that active vitamin D is able to suppress cell proliferation [37]. Laboratory and preclinical studies have also reported that vitamin D inhibits carcinogenesis, decreases tumor invasiveness and the propensity to metastasize [38]. Positive results in human studies are mainly derived from post hoc analyses, secondary end-points or meta-analyses. The last show not a decrease in cancer incidence but rather in mortality [39]. We must therefore proceed with a word of caution. Until it has been clearly demonstrated that there is a causal relationship, these positive “non-primary, end-point results” should be considered as a background for generating new hypotheses for future investigations.

## 4. Conclusions

The field of vitamin D is rapidly evolving. We believe that in the near future at least some of today’s uncertainties may be replaced by established certainties. Some steps forward have been implemented. In this context, the standardization of 25(OH)D assays will clarify uncertainties deriving from inconsistent results. The completion of studies with the enrolment of thousands of participants addressing the skeletal but mainly the non-skeletal effects of vitamin D will also clarify a number of issues. Some of these studies are beginning to be published [40,41]. Regarding the two specific aspects covered in this review, insights into the long-lasting debate concerning the optimal vitamin D regimen might be achieved once, for example, the role of free vitamin D with respect to the total amount is better clarified. At the same time, health policy promoting vitamin D fortification and biofortification, together with adequate lifestyle changes, will decrease the number of subjects with hypovitaminosis in the world. These policies have been demonstrated to be possible and successful. Long-term studies with vitamin D in patients with documented hypovitaminosis D will better address its role in cancer prevention.

## Figures and Tables

**Table 1:** Most important (and unsolved) controversies surrounding Vitamin D.

Standardization of the methods employed to measure 25-hydroxyvitamin D (25(OH)D), its precursors and metabolites Which metabolite to measure: one or multiple? The role of free vs. total 25(OH)D measurement Definition of hypovitaminosis D (insufficiency, deficiency) Definition of vitamin D toxicity (and corresponding threshold) Screening vs. treatment Threshold for hypovitaminosis in general population vs. specific clinical condition (i.e., pregnancy, lactation) and diseases states (i.e., kidney failure, primary hyperparathyroidism, glucocorticoid-induced osteoporosis)
• Modalities to reach vitamin D sufficiency ✓ UVB ✓ Food ✓ Fortification ✓ Bio-fortification ✓ Supplements
• Modalities to reach vitamin D sufficiency with cholecalciferol ✓ Daily ✓ Weekly ✓ Monthly ✓ Intermittently
• Which vitamin D should be utilized? ✓ Ergocalciferol ✓ Cholecalciferol ✓ Calcidiol ✓ Calcitriol ✓ 1α(OH)D ✓ Other metabolites
Vitamin D supplementation: Effects on bone mineral density Vitamin D supplementation: Effects on fractures Vitamin D supplementation: Effects on falls Vitamin D supplementation and extra-skeletal effects
✓ Cancer ✓ Neurological diseases ✓ Immunological diseases ✓ Cardiovascular diseases ✓ Infectious diseases ✓ ………………….

**Table 2:** Main features of studies addressing vitamin D repletion in normal subjects.

Authors	Population	Treatment	Aim	Study	Controls	Basal Value (ng/mL)	Final Value (ng/mL)	Duration
Pietras S. et al. [20]	Subjects 18 years or older	D_2_ 50,000 I.U (weekly for 8 weeks and every other week)	Prevent recurrence of vitamin D deficiency and maintain adequate levels in vitamin D sufficient subjects	Retrospective	No	23.4	47	5–72 months (mean 26)
Chel V. et al. [21]	Elderly nursing home residents (84.3 ± 6.3 years)	D_3_ 600 I.U./die	Efficacy of different doses and intervals in reaching vitamin D sufficiency	Randomized vs. placebo	Yes	9.2	28	4 months
Chel V. et al. [21]	Elderly nursing home residents (84.3 ± 6.4 years)	D_3_ 4200 I.U. weekly	Efficacy of different doses and intervals in reaching vitamin D sufficiency	Randomized vs. placebo	Yes	11	27	4 months
Chel V. et al. [21]	Elderly nursing home residents (83.9 ± 6.9 years)	D_3_ 18,000 I.U./monthly	Efficacy of different doses and intervals in reaching vitamin D sufficiency	Randomized vs. placebo	Yes	9.5	21	4 months
Meyer O. et al. [22]	Healthy postmenopausal women (63.4 ± 7.8 years; *n* = 10)	D_3_ 800 I.U./day or 5600 I.U. weekly	Efficacy in increasing 25(OH)D circulating levels	Randomized double-blind	No	14.2	31	4 months
Tripkovic L. et al. [23]	Women 20–64 years (44.3 ± 11.18; *n* = 67)	D_2_ 600 I.U.	Increase 25(OH)D levels in winter	Randomized double-blind	Yes	18	24	12 weeks
Tripkovic L. et al. [23]	Women 20–64 years (43 ± 12.73; *n* = 67)	D_3_ 600 I.U.	Increase 25(OH)D levels in winter	Randomized double-blind	Yes	17	29.5	12 weeks
